# Alcohol and Tobacco Content in UK Video Games and Their Association with Alcohol and Tobacco Use Among Young People

**DOI:** 10.1089/cyber.2016.0093

**Published:** 2016-07-01

**Authors:** Jo Cranwell, Kathy Whittamore, John Britton, Jo Leonardi-Bee

**Affiliations:** UK Centre for Tobacco and Alcohol Studies, Division of Epidemiology and Public Health, University of Nottingham, Nottingham, United Kingdom.

## Abstract

To determine the extent to which video games include alcohol and tobacco content and assess the association between playing them and alcohol and smoking behaviors in adolescent players in Great Britain. Assessment of substance in the 32 UK bestselling video games of 2012/2013; online survey of adolescent playing of 17 games with substance content; and content analysis of the five most popular games. A total of 1,094 adolescents aged 11–17 years were included as participants. Reported presence of substance content in the 32 games; estimated numbers of adolescents who had played games; self-reported substance use; semiquantitative measures of substance content by interval coding of video game cut scenes. Nonofficial sources reported substance content in 17 (44 percent) games but none was reported by the official Pan European Game Information (PEGI) system. Adolescents who had played at least one game were significantly more likely ever to have tried smoking (adjusted odds ratio [OR] 2.70, 95 percent confidence interval [CI] 1.75–4.17) or consumed alcohol (adjusted OR 2.35, 95 percent CI 1.70–3.23). In the five most popular game episodes of alcohol actual use, implied use and paraphernalia occurred in 31 (14 percent), 81 (37 percent), and 41 (19 percent) intervals, respectively. Tobacco actual use, implied use, and paraphernalia occurred in 32 (15 percent), 27 (12 percent), and 53 (24 percent) intervals, respectively. Alcohol and tobacco content is common in the most popular video games but not reported by the official PEGI system. Content analysis identified substantial substance content in a sample of those games. Adolescents who play these video games are more likely to have experimented with tobacco and alcohol.

## Introduction

In 2015, the UK video gaming market was estimated to be worth €5.8 billion, the third biggest in the world.^1,2^ Although around 54 percent of UK adolescents play video games online,^3^ parental concern over exposure to inappropriate content while playing video games appears to be lower than for other media.^3^ While 80 percent of children age 10–15 are estimated to play packaged or online video games with an age rating higher than their age, over half of British parents are unaware of the content this exposes them to.^4^

Age classification of UK video games is the responsibility of the Video Standards Council, which applies age ratings based on content descriptors assigned by the Pan European Game Information (PEGI) system to help parents decide whether game content is suitable for their children.^5,6^ The PEGI system is self-regulatory and voluntary, and age ratings are assigned based on a content declaration form submitted by the game developer. PEGI does not check the content of game play against these statements.^7^ Approximately 293 industry signatories across 28 countries use the PEGI system.^5^

Adolescents who are exposed to tobacco or alcohol content in films are more likely to become smokers or to use alcohol than those who are not,^8–15^ and for tobacco at least this association is causal.^16^ Tobacco and alcohol content is highly prevalent in a range of other popular media,^2,17–22^ and the interactive nature of video games provides multiple opportunities to promote products and behaviors.^23–25^ However, the extent to which video games expose young people to tobacco or alcohol imagery has not been widely investigated.^16,26^ This study aims to quantify and characterize alcohol and tobacco content in the most popular UK video games, and to assess the relationship between exposure to tobacco or alcohol content and smoking or drinking behaviors in British adolescents.

## Methods

### Procedure

#### Reported tobacco and alcohol content in popular games

We used published top-100 UK game popularity charts for 2012 and 2013^27–29^ to identify all 39 video games in the eight manufacturer-defined games genres of stealth, stealth/action adventure, stealth/open world, stealth/shooter, shooter, shooter/open world, open world, and survival/horror because they involve avatars that look and act like real people. For the 39 identified games, we searched for reported presence or absence of alcohol and tobacco content in the official content descriptors provided by the PEGI,^6^ the official game developer Web sites, Amazon.co.uk^30^ and game.co.uk^31^ retail Web sites; and the Internet Movie Database (IMDb)^32^ and Common Sense Media (CSM)^33^ Web sites, which provide information and reviews on a variety of media, including video games. All content descriptors from these sources were recorded, including parental advice in the form of user reviews from IMDb and CSM. Tobacco or alcohol content was verified in at least one of these sources for 17 of the 39 selected games (Table 1).

**Table 1.** Sources Indicating the Presence of Either Alcohol or Tobacco Content Among 39 Genre-Eligible Top Video Games for 2012 and 2013

				*Sources indicating presence of alcohol or tobacco content, including examples*	
*Game title*	*Publisher*	*PEGI age rating*	*Year*	*Alcohol content*	*Tobacco content*
Call of Duty: Black Ops II	Activision Blizzard UK	18	2012	IMDb “There is some alcohol consumption and smoking in the game”	IMDb “There is some alcohol consumption and smoking in the game”
Hitman: Absolution	Square Enix Ltd.	18	2012	IMDb “You go to a club in one mission called the ‘Vixen Club.’ You see people drinking and there is a bar”	—
Far Cry 3	Ubisoft EMEA	18	2012	Common Sense Media “Parents need to know that Far Cry 3 has a lot of mature content, ranging from excessive violence and blood to sexual imagery (including a brief love-making session) as well as drug and alcohol references and strong profanity.”	—
Borderlands 2	Take2 Interactive Software Europe Ltd.	18	2012	IMDb, Common Sense Media “One character is an alcoholic” “The game has sexual references, alcohol consumption, and profanity”	—
Mass Effect 3	EA Swiss Sarl	18	2012	IMDb “One of the main characters, Ashley is shown lying on the floor at one point with a hangover. Later, if she is present in the game Tali will be seen attempting to get drunk at the ship's bar. In the Citadel DLC pack, Ashley and Shepard (if the two characters are romanced) will engage in a drinking game where they take several shots of wine. They do end up slightly drunk, but not excessively. In the same DLC, if they are present and depending on the choices made by the player, the characters of Tali and Grunt may be found completely drunk in one of the bathrooms”	—
Battlefield 3	EA Swiss Sarl	16	2012	—	IMDb “There is some smoking, but It is all brief”
Call of Duty: Modern Warfare 3	Activision Blizzard UK	18	2012	—	IMDb, Common Sense Media One of the characters, Captain Price smokes a cigar at some points. At the end of the game, he is shown lighting one up and inhaling it from a first-person-perspective. In stealth missions, you will see enemies smoking like the mission “Eye Of The Storm” and “Stronghold” “Soldiers use strong language, and one of them is a frequent cigar smoker”
Max Payne 3	Take2 Interactive Software Europe Ltd.	18	2012	IMDb, Common Sense Media, Developer “Max Payne is addicted to painkillers. We see him taking pills and washing them down with hard alcohol, or simply drinking alcohol in more than half of the cutscenes.” It deals with serious issues including depression, alcoholism, and loss.” “The game contains frequent alcohol use by the main character”	IMDb “Max and other characters can infrequently be seen smoking”
Sleeping Dogs	Square Enix Ltd	18	2012	IMDb, Common Sense Media “Many drug and alcohol references exist in the storyline.” “There is also a lot of profanity, sexual innuendo, and imagery tied to consuming drugs and alcohol”	—
Tom Clancy's Ghost Recon: Future Soldier	Ubisoft EMA	18	2012	IMDb “You see army men drink beer in the cafeteria, some also smoke”	IMDb “You see army men drink beer in the cafeteria, some also smoke”
Grand Theft Auto V	Rockstar Games	18	2013	IMDb, Common Sense Media, Developer “Players have the option to drink beer and get drunk, which will blur their vision and make them less stable.” “Parents need to know that Grand Theft Auto V is an M-rated action game brimming with … drug and alcohol abuse. Players also have the opportunity to make their avatars use marijuana and drink alcohol, both of which impact their perception of the world.” “Use of drugs and alcohol”	IMDb “Several characters smoke joints and cigarettes throughout the game”
Grand Theft Auto Episodes: Liberty City	Rockstar Games	18	2012	Developer “Use of drugs and alcohol”	—
Grand Theft Auto IV	Rockstar Games	18	2012	IMDb, Common Sense Media, Developer “Players can become intoxicated and drive. The game penalizes the player for doing so by making it difficult to steer and drive, and any nearby police will also converge on the player if this attempted. The option to hail a taxi is given, but due to inability to walk properly, it is made very difficult to actually do this.” “This new version is as controversial as its predecessors, letting you lead a life of crime, shoot police officers, drink and drive, and have sex with prostitutes.” “Use of drugs and alcohol”	IMDb “This game takes place around the same time that the smoking ban in New York City was first being implemented. One of the characters in-game ignores the law and smokes cigarettes in a bar and numerous characters make dialogue references to this”
Assassin's Creed IV: Black Flag	Ubisoft EMA	18	2013	IMDb “Edward can drink ale, and the screen will get foggy and eventually he will pass out and wake up in a different location”	—
Batman: Arkham Origins	Warner Brothers Entertainment UK Ltd.	16	2013	IMDb “Frequent smoking, and some drinking”	IMDb “Frequent smoking, and some drinking”
BioShock Infinite	Take2 Interactive Software Europe Ltd.	18	2013	IMDb, Common Sense Media “Occasionally alcohol can be used to regain health, the player can also smoke.” “Action sequences see player avatars dismembering enemies in gruesome fashion, drinking and smoking to restore health and special abilities”	IMDb, Common Sense Media “Occasionally alcohol can be used to regain health, the player can also smoke” “Action sequences see player avatars dismembering enemies in gruesome fashion, drinking and smoking to restore health and special abilities”
Assassin's Creed III	Ubisoft EMA	18	2013	IMDb “Some characters drink alcohol in a few scenes”	—

— indicate no mention of content.

IMDb, Internet Movie Database; PEGI, Pan European Game Information.

#### Adolescent game content exposure, and alcohol and tobacco use

Through a *YouGov* Omnibus online survey carried out between October 23 and November 3, 2014, we asked British adolescents aged 11–17 years whether they had played any of the 17 most popular video games identified as containing either tobacco or alcohol imagery. YouGov is an organization that conducts online surveys of representative samples of the UK population. In accordance with *YouGov* practice, adolescents aged 16–17 were recruited by direct e-mail invitations to a random sample of panelists from a database of individuals who had consented to be contacted, informing them of the survey and inviting them to take part; and adolescents aged 11–15 were recruited by e-mailing parents or legal guardians from the *YouGov* database and asking them, after reading the study information, to explain the nature of the study and what was required, and request oral assent of the young person. Consenting respondents then followed a URL link to complete the online survey.

The game play questions included: “Which of these video games have you ever played?” and, for those who had played at least one game, “You said that you had played these video games [games listed], which of these video games have you ever completed at least half of?” All respondents were then asked “How often do you play video games that are recommended for people aged 18 or over?” Two questions about smoking and alcohol behavior using the question “Which ONE of the following BEST applies to you?” For smoking behavior, the possible responses were (1) I have never smoked cigarettes, not even a puff or two, (2) I have only ever tried smoking cigarettes once, (3) I used to smoke sometimes, but I never smoke cigarettes now, (4) I sometimes smoke cigarettes now, but less than one a week, (5) I usually smoke between one and six cigarettes a week, (6) I usually smoke more than six cigarettes a week, or (7) Don't want to say. For alcohol behavior, the possible responses were (1) I have never drank alcohol, not even a sip or two, (2) I have only ever tried drinking alcohol once, (3) I used to drink alcohol sometimes, but I never drink now, (4) I sometimes drink alcohol, but less than once a week, (5) I usually drink alcohol once a week or more often, or (6) Don't want to say. The *YouGov* survey was carried out in accordance with both the MRS (Market Research Society) and the BPC (British Polling Council) codes of ethics and practice.

#### Content analysis of the five most popular video games

To provide more detail of the nature of alcohol and tobacco content in these games, we selected the five games identified to be most popular in our survey results for a content analysis of published game “cut scenes.” Analyzing the content seen by individual players is logistically difficult, in part, because of the amount of time spent playing and, in part, because each player's experience of the game is different and hence involves different exposure. We therefore limited our analysis to the “cut scenes” designed to provide a narrative to the game storyline and occur when a player moves up a level or when a character dies, and hence are seen by most players. We searched the video sharing Web site *YouTube* using the name of the game followed by the key term “full cut scene movie.” *YouTube* videos can be uploaded by any account holder at any time. Although YouTube will sometimes remove a video that is offensive or otherwise inappropriate, *YouTube* content is generally uncontrolled, and in accordance with usual practice for social networking sites, those uploading the videos can remain anonymous to the viewer. We therefore had no control over the selection of cut scenes from video games on the site. However, to ensure we captured as many cut scenes as possible, our inclusion criteria included uploads that (a) had the longest run time, (b) claimed to include all cut scenes, and (c) excluded any game play.

Games were analyzed for alcohol and tobacco content using the semiquantitative interval coding method previously described for films.^20^ We analyzed the visual and audio content of the games using 5-minute intervals, coding each for the presence or absence of alcohol, tobacco, or electronic cigarettes in the following categories: actual use, implied use without actual use, paraphernalia without actual or implied use, and brand appearance (real or fictitious), and *any* alcohol or tobacco content (any of the above). Characters who consumed alcohol were coded for obvious inebriation and for appearing as either adults or children for both alcohol and tobacco use. The different types of alcohol and tobacco occurring in each coding interval, and the social context of use, were also recorded.

Authors J.C. and K.W. carried out the coding independently following a pilot of one game to assess consistency in coding. Any discrepancies were discussed and resolved. Repeated appearances in the same category during any single 5-minute interval were coded as a single event and appearances in different categories as separate events, with the exception of brands, for which different brands were counted as separate events. Where different categories of appearance of alcohol, tobacco, or electronic cigarettes occurred simultaneously (e.g., actual and implied use of alcohol), the episode was coded under the higher of the categories as ranked above. Any partial intervals at the end of a video were counted as a full 5-minute interval.

### Data analysis

Survey data were summarized as weighted percentages for categorical variables and weighted mean with standard error for continuous variables, with weights derived based on age, sex, and government region of residence. Chi-squared tests were used to assess the association between categorical variables. We used multiple logistic regression to estimate age- and sex-adjusted odds ratios (ORs) with 95 percent confidence intervals (CIs). *p* Values <0.05 were deemed statistically significant. Data were analyzed using Stata MP 13.1 for Windows (StataCorp LP, College Station, TX).

## Results

Of the 39 eligible games, content descriptors for 17 (44 percent) included either tobacco (10 games) or alcohol (13 games; Table 1). All but two of these 17 games (Battlefield 3 and Batman: Arkham Origins, both age rated 16 years) were PEGI, age rated 18 years. Alcohol and tobacco content was disclosed for all 17 films on the IMDb, CSM, and developer Web sites, but was not mentioned by PEGI, Amazon, or Game.co.uk (Table 1).

### Survey

A total of 1,094 adolescents aged 11–17 years responded to the survey. The mean age of the respondents was 14 years (standard deviation 1.9) and 50 percent were male. The majority of respondents resided in the North of England (26 percent), the South of England (23 percent), or the Midlands (19 percent). One thousand eighty-two (99 percent) respondents provided information on video games played. Sixty percent of adolescents had played at least one of the 17 video games containing tobacco or alcohol content and were more likely to be male than female (78 percent versus 41 percent; *p* < 0.001) and to be older (15–17 years, 68 percent; 11–14 years, 53 percent; *p* < 0.001).

The average number of video games played that contained content on tobacco and alcohol was 4.6 (95 percent CI 4.3–4.9), with similar numbers of video games being played by older and younger adolescents (11–14 years: mean 4.5, 95 percent CI 4.1–4.8; 15–17 years: mean 4.7, 95 percent CI 4.3–5.1) (*p* = 0.4). The average number of video games played was significantly greater in males (mean 5.2, 95 percent CI 4.9–5.5) compared to females (mean 3.4, 95 percent CI 3.0–3.8) (*p* < 0.001). More than two-thirds of adolescents who had played at least one game had completed at least half of the game(s) played (70 percent), with similar proportions by age group (11–14 years: 71 percent, 15–17 years: 69 percent; *p* = 0.6). However, significantly more males reported completing at least half of the game(s) than females (males: 79 percent, females: 51 percent; *p* < 0.001).

Sixty percent of adolescents had played a game rated 18+ years, with significant differences by age group (11–14 years: 55 percent, 15–17 years: 67 percent; *p* < 0.001) and by gender (males: 75 percent, females: 44 percent; *p* < 0.001). Twenty-eight percent of adolescents played games with an age rating of 18+ years at least once a week, with similar proportions by age group (11–14 years: 29 percent, 15–17 years: 27 percent; *p* = 0.5). However, significantly more males reported playing games with an age rating of 18+ years at least once a week than females (males: 42 percent, females: 13 percent; *p* < 0.001).

#### Ever tried smoking

Overall, 6 percent of adolescents declared themselves as current smokers, 2 percent were ex-smokers, and 9 percent had tried smoking only once, and thus, 17 percent were coded as ever smokers. In univariable analyses, the odds of ever trying smoking were significantly associated with playing at least one of the 17 games and ever playing video games rated 18+ years (Table 2). After adjustment for age and sex, the odds of ever trying smoking were significantly associated with playing at least one of the video games (OR 2.70, 95 percent CI 1.75–4.17), completing at least half of the video games (OR 1.72, 95 percent CI 1.02–2.90), and ever playing a video game rated 18+ years (OR 4.08, 95 percent CI 2.57–6.47).

**Table 2.** Independent Effects of Playing Video Games Which Include Tobacco and/or Alcohol Content on the Odds of Ever Tried Smoking or Ever Tried Alcohol

	*Crude OR (95% CI)*	*Adjusted OR*^a^*(95% CI)*
Ever tried smoking		
Played at least one of the video games	2.56 (1.73–3.78)	2.70 (1.75–4.17)
Completed at least half of the video games	1.22 (0.77–1.95)	1.72 (1.02–2.90)
Ever played video game rated 18+ years	3.50 (2.25–5.43)	4.08 (2.57–6.47)
Played video games rated 18+ years at least once per week	1.06 (0.72–1.55)	1.29 (0.83–2.01)
Ever tried alcohol		
Played at least one of the video games	2.27 (1.73–3.00)	2.35 (1.70–3.23)
Completed at least half of the video games	0.83 (0.55–1.27)	0.97 (0.61–1.53)
Ever played video game rated 18+ years	2.56 (1.93–3.38)	2.68 (1.95–3.68)
Played video games rated 18+ years at least once per week	1.40 (1.02–1.91)	1.65 (1.17–2.34)

^a^ Adjusted for age and sex.

CI, confidence interval; OR, odds ratio.

#### Ever tried alcohol

The results show that 36 percent of adolescents reported as current alcohol drinkers, 4 percent were ex-drinkers, and 31 percent had tried drinking alcohol only once, and thus, 71 percent were coded as ever tried alcohol. In univariable analyses, the odds of ever trying alcohol were significantly associated with playing at least one of the 17 games, ever playing video games rated 18+ years, and playing video games rated 18+ years at least once per week (Table 2). After adjustment for age and sex, the odds of ever trying alcohol were significantly associated with playing at least one of the video games (OR 2.35, 95 percent CI 1.70–3.23), ever playing a video game rated 18+ years (OR 2.68, 95 percent CI 1.95–3.68), and playing video games rated 18+ years at least once per week (OR 1.65, 95 percent CI 1.17–2.34).

#### Co-addictions of smoking and alcohol

Twenty-nine percent of adolescents had never tried smoking or alcohol, 54 percent had tried only alcohol, 1 percent had tried only smoking, and 16 percent had tried both alcohol and smoking. After adjustment for age and sex, adolescents who had ever tried smoking *and* alcohol were five times more likely to have played at least one of the 17 video games with tobacco or alcohol content (OR 4.94, 95 percent CI 2.96–8.26) compared to adolescents who had never tried smoking *nor* alcohol. A doubling in odds of playing at least one of the 17 video games was seen in adolescents who either had only tried alcohol (OR 2.11, 95 percent CI 1.51–2.94) *or* only tried smoking (OR 2.78, 95 percent CI 0.49–15.74), although the latter association was not statistically significant due to a lack of power from small frequencies.

### Content analysis of the top five games

The survey results identified the most popularly played games to be Call of Duty: Black Ops II, Grand Theft Auto V, Call of Duty: Modern Warfare 3, Grand Theft Auto IV, and Assassin's Creed III, which had been viewed, respectively, by 39 percent, 34 percent, 36 percent, 30 percent, and 23 percent of adolescents aged 11–17 years. We identified cut scene videos for all of these games, comprising a total of 1,075 minutes of screen time (mean per game 215 minutes, range 113–288 minutes) and 217 five-minute intervals for analysis.

#### Alcohol content

**FIG. 1.**
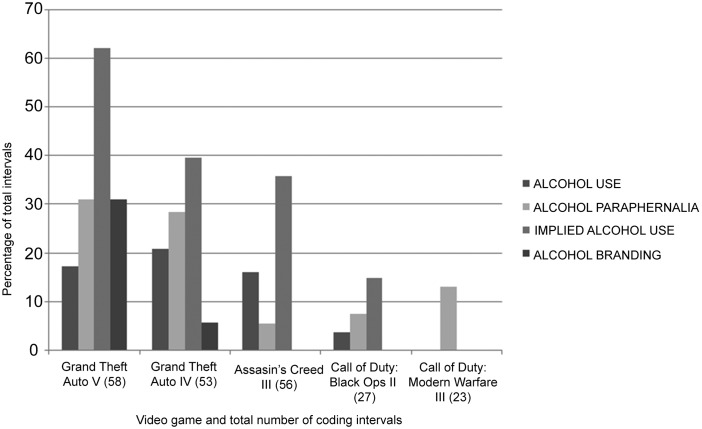
Percentage of total coding intervals containing alcohol content by category in the five games.

Alcohol content occurred in all five games, in a total of 103 intervals (48 percent of all coding intervals) and highest in *Grand Theft Auto V* and *IV.* Episodes of actual alcohol use and implied use occurred in four games, and in 31 (14 percent) and 81 (37 percent) of all coding intervals, respectively. *Modern Warfare III* had no alcohol content in these categories. Obvious inebriation was visible in five (16 percent) and six (7 percent) of actual and implied use intervals, respectively. All alcohol users appeared to be adults and all were male. Episodes of alcohol paraphernalia occurred in 41 (19 percent) intervals and in all five games, and alcohol branding in 21 (10 percent) intervals in two games (both Grand Theft Auto games, 18 intervals in Grand Theft Auto V alone) but not in any others. All identified brands (Pißwasser Lager, Logger Beer, Dusche Gold Beer, Richard's Whiskey, Benedict Beer, Cerveza Barracho, The Mount Whiskey, Jakey's Whiskey, and Cherenkov Vodka) were fictitious. Details of alcohol content by category for each video game are given in Figure 1. Alcohol users in the games were just as likely to drink alone as with other users or with other nonalcohol users (12, 10, and 9 in each category, respectively). The types of alcohol consumed were mainly beer (7 intervals) and spirits (13 intervals), however, unknown drinks were consumed in GTA IV and Assassin's Creed (12 intervals).

#### Tobacco

**FIG. 2.**
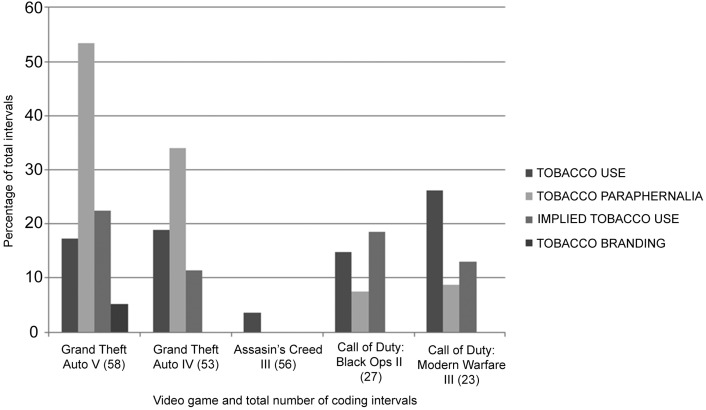
Percentage of total coding intervals containing tobacco content by category in the five games.

Tobacco content occurred in all five games, in 79 (36 percent) intervals and was highest in *Grand Theft Auto V* and *IV*. Episodes of actual tobacco use occurred in 32 (15 percent) of all coding intervals. All tobacco users appeared to be adults and 88 percent were male. Implied use appeared in 27 (12 percent) of all coding intervals and tobacco paraphernalia occurred in 53 (24 percent) intervals in all games apart from *Assassin's Creed III*. Tobacco branding only occurred once in the game Grand Theft Auto V and involved the fictitious Redwood brand.^34^ Details of tobacco content by category for each game are given in Figure 2. Most tobacco smoking occurred with other, nontobacco users rather than alone or with other tobacco users (25, 4, and 2 in each category, respectively). One other occurrence was identified in a photo of a man smoking a cigar. There was no electronic cigarette content.

## Discussion

This is the first study to examine the relationship between playing games with alcohol and tobacco content and related behaviors in British adolescents; and the first to quantify alcohol and tobacco content in bestselling UK video games involving human avatars. Our findings demonstrate that alcohol and tobacco content is common, appearing in all five of the top most played games from the survey and occurring, respectively, in nearly half and over a third of all coding intervals. We also demonstrate that young people exposed to this content are more than twice as likely to have used tobacco or alcohol. Our findings also demonstrate that the PEGI system does not identify alcohol or tobacco content in games, at all in our sample. Other researchers have attempted to examine the relationship between general video game playing and smoking with no clear conclusions as it was not the primary focus or was beset by methodological issues (see Forsyth and Malone^26^ for a detailed review). Our research is new because it specifically examines the association between playing videos games with smoking and related smoking behaviors. To our knowledge, no other study has directly examined the relationship between alcohol in video games and alcohol-related behaviors in adolescents.

Since we relied on external sources to identify our sample of games containing tobacco or alcohol imagery, it is possible that we have underestimated the proportion of games, including this content. Furthermore, because we did not include actual game play in our analysis of the top five games, we are almost certainly underestimating the likely total smoking and drinking content in these games. We also acknowledge that exposure to smoking and drinking could be moderated by individual experiences of game play and time spent playing particularly in games that include role play where the players' choices impact on the story and plot, thus resulting in more or less exposure. All observed alcohol and tobacco brands were fictitious, and so, no industry advertising codes were violated.

Although coverage bias is problematic for most survey methods, we used YouGov because it draws sufficiently from lower prevalence groups and because online surveys provide social distance, which reduces respondent reluctance to reveal sensitive information.^35^ The cross-sectional design and the limited potential confounders collected in our study limit the validity of causal inference from our findings. However, exposure to tobacco and alcohol in other media encourages uptake of drinking independently of other factors,^11,12^ so it is plausible that exposure to tobacco or alcohol content in the games also encourages these behaviors. Furthermore, recent research suggests that tobacco content in video games is recalled consciously by players, which suggests some level of content-related cognitive processing during game play.^36^

Our findings are consistent with those of Forsyth and Malone,^36^ who found that The Entertainment Software Ratings Board (ESRB), the US equivalent of the PEGI system, included tobacco content descriptors in only 4 percent of the 75 percent of M-rated games (suitable for those aged older than 17 years) verified by the authors as having tobacco content. Similarly, in our study, we found that PEGI included no tobacco or alcohol content descriptors for any of the games we verified as including content; unlike the ESRB system, the PEGI system does not have any policy to provide content descriptors for either alcohol or tobacco. The main way we verified content was through the parental advice sections of IMDb (found in in16/17 games) and CSM (in 7/17 games), which suggests that this is something that parents want to know about, but neither of which are the natural first port of call for parents.

Video games are clearly attractive to adolescents regardless of age classification. It appears that official PEGI content descriptors are failing to restrict youth access to age inappropriate content because they are not an accurate indicator of the full range of content such as tobacco and alcohol. Although the system only rates around 4 percent of games as suitable for adults aged 18 years or older, our findings demonstrate that 60 percent of our survey adolescents had played at least one of the 17 video games, 15 of which are intended for adult use. We therefore suggest that the PEGI system needs to include both alcohol and tobacco in the content descriptors. In addition, game developers could be offered incentives to reduce the amount of smoking and drinking in their games or to at least reference smoking and drinking on their packaging and Web sites. As a child protection method, it is naive for both the games industry and the Interactive Software Federation of Europe, who regulate the PEGI system, to rely on age ratings alone. Future research should focus on identifying the levels of exposure in terms of dose that youth gamers are exposed to during actual game play and the effects of this on long-term alcohol and smoking behavior.
